# The effects of transcutaneous auricular vagus nerve stimulation in epilepsy comorbid with migraine on the EEG power spectrum: a randomized controlled trial

**DOI:** 10.3389/fneur.2025.1694455

**Published:** 2025-12-12

**Authors:** Shuai Ma, Jinyuan Du, Cheng Luo, Xiao Wu, Linli Liu, Tingting Liu, Xin Zhang, Lili Yang, Jie Liu, Yi Luo, Sheng Zhang, Roberto Rodriguez-Labrada, Qiong Zhu

**Affiliations:** 1 Department of Neurology, Sichuan Provincial People’s Hospital, University of Electronic Science and Technology of China, Chengdu, China; 2 China-Cuba Belt and Road Joint Laboratory on Neurotechnology and Brain-Apparatus Communication, School of Life Science and Technology, The Clinical Hospital of Chengdu Brain Science Institute, University of Electronic Science and Technology of China, Chengdu, China; 3 Department of Neurology, Enyang district Peoples Hospital of Bazhong, Bazhong, Sichuan, China; 4 Cuban Neuroscience Center, La Habana, Cuba

**Keywords:** transcutaneous auricular vagus nerve stimulation, epilepsy, migraine, comorbidity, EEG power spectrum

## Abstract

**Background:**

Migraine is a common comorbidity in patients with epilepsy, with a comorbidity rate ranging from 9.3 to 34.7%. Transcutaneous auricular vagus nerve stimulation (taVNS) is an emerging therapy used in both epilepsy and migraine treatment. However, there are currently no randomized controlled studies (RCTs) using taVNS for epilepsy complicated with migraine.

**Objective:**

In this study, we evaluated the effect of taVNS as an adjuvant therapy on patients with comorbid epilepsy and migraine.

**Methods:**

Forty comorbid patients (taVNS *n* = 20, tanVNS *n* = 20) were recruited and randomly grouped. The taVNS group received the true stimulus, whereas the tanVNS group received a pseudostimulus. Outcome assessment was performed at baseline and 24 weeks after initiation. We used *t*-test and non-parametric tests to analyse the data.

**Results:**

The frequencies of migraine attacks and seizures significantly decreased in the taVNS group from baseline to 24 weeks (migraine attack frequency, *p* = 0.002; seizure frequency, *p* = 0.004), and so did in Self-Rating Anxiety Scale (SAS) score (*p* < 0.001) and Self-Rating Depression Scale (SDS) score (*p* < 0.001). The QOLIE-31 scores increased after 24 weeks of taVNS treatment (*p* = 0.028). Moreover, taVNS reduced the EEG power spectrum in four frequency bands at 16 electrode locations in comparison between groups (*p* < 0.05).

**Conclusion:**

In comorbid patients in our groups, taVNS can decrease the frequency of seizures, improve mood and quality of life, and reduce the EEG power spectrum.

## 1 Introduction

Epilepsy and migraine are both common neurological disorders with paroxysmal, chronic and recurrent clinical manifestations (1). Epilepsy is characterized by recurrent seizures, which are brief episodes of involuntary movement that may involve a part of the body (partial) or the entire body (generalized) and are sometimes accompanied by loss of consciousness and control of bowel or bladder function (1). Migraine attacks are mainly unilateral pulsatile headaches, and are sometimes accompanied by nausea and vomiting (1). In particular, migraine is one of the most common comorbid neurological conditions in patients with epilepsy (2–6). Epidemiological surveys have shown that the incidence of migraine in epilepsy patients ranges from 9.3 to 34.7% (7–9), moreover, it has been suggested that migraine, a common comorbidity in patients with epilepsy, exacerbates epilepsy, and vice versa (10–14). Therefore, patients with comorbid epilepsy and migraine suffer long-term pain, and their quality of life is reduced.

Vagus nerve stimulation (VNS) has been approved by the FDA as a treatment for drug-resistant epilepsy in patients aged >12 years, and it is also used to treat migraine (15–17). However, the electrode implantation device used in traditional VNS may cause some inconveniences, such as surgical wound infection and rejection reactions, and the cost of therapy is high (18). To improve cost-effectiveness and reduce invasiveness, transcutaneous auricular vagus nerve stimulation (taVNS) was developed on the basis of traditional VNS (19, 20). Instead of the implantation of a stimulator in the chest, taVNS involves wearing a lightweight stimulator in the concha auricula to stimulate the auricular branch of the vagus nerve (ABVN), which can ameliorate seizures and migraines (19, 20). The auricular concha region is innervated mainly by the ABVN, which is often termed Alderman’s nerve or Arnold’s nerve. Previous studies have shown that the ABVN can project directly to the nucleus of the solitary tract (NTS) and then further connect with other brain regions, such as the locus coeruleus (LC), thalamus, and prefrontal cortex (21–25). Moreover, taVNS can modulate cortical networks including the default mode network (DMN), salience network, and central executive network, among which the DMN has been the most studied (26–32).

TaVNS has been shown in clinical studies to alleviate epilepsy and migraine. A meta-analysis and systematic review of nine clinical studies (including 788 patients with epilepsy) and eight preclinical studies revealed that taVNS was effective in treating patients with epilepsy (33). In 59 migraine patients, both pain intensity and migraine attack frequency were significantly reduced after 4 weeks of taVNS treatment (34). In this study, we applied taVNS as an adjunctive therapy for comorbid epilepsy and migraine and evaluated its effects. Electroencephalograms (EEGs) are widely used to monitor epilepsy and migraine patients, and the EEG power spectrum can be used to evaluate the effect of VNS (35). Power spectrum analysis assumes that the EEG is a linear combination of simple vibrations at a specific frequency, and involves the decomposition of each frequency component of the EEG signal to determine its magnitude (or power) (35). Therefore, we use EEG power spectrum analysis as one of the approaches in this study.

By comparing the frequency of epileptic seizures and migraine attacks, Self-Rating Anxiety Scale (SAS) and Self-Rating Depression Scale (SDS) scores, and quality of life scores, we evaluate the effect of taVNS as an adjuvant therapy in patients with comorbid epilepsy and migraine. By analysing the EEG power spectrum, we explore changes in different frequency bands after taVNS treatment in an attempt to identify potential evaluation indicators.

## 2 Method

### 2.1 Study design

This randomized controlled trial included patients who were admitted to the hospital from September 2023 to June 2024. The protocol was approved by the Clinical Trial Ethics Committee of Sichuan Provincial People’s Hospital (Number 430, 2023). Informed consent was obtained from all the patients.

In a previous RCT of patients with only epilepsy, after 24 weeks of treatment with taVNS, 8/47 patients were seizure free, and 19/47 patients had a reduced seizure frequency (36). Referring to it, we chose a sample size of approximately 42 comorbid patients to achieve a power of 0.80 and a standardized effect size of 0.6 (seizure frequency: times/month). Patients with comorbid epilepsy and migraine were randomly allocated to the true stimulation group (taVNS group) or the pseudostimulation group (transcutaneous auricular nonvagus nerve stimulation (tanVNS) group) at a 1:1 ratio.

**Figure 1:**
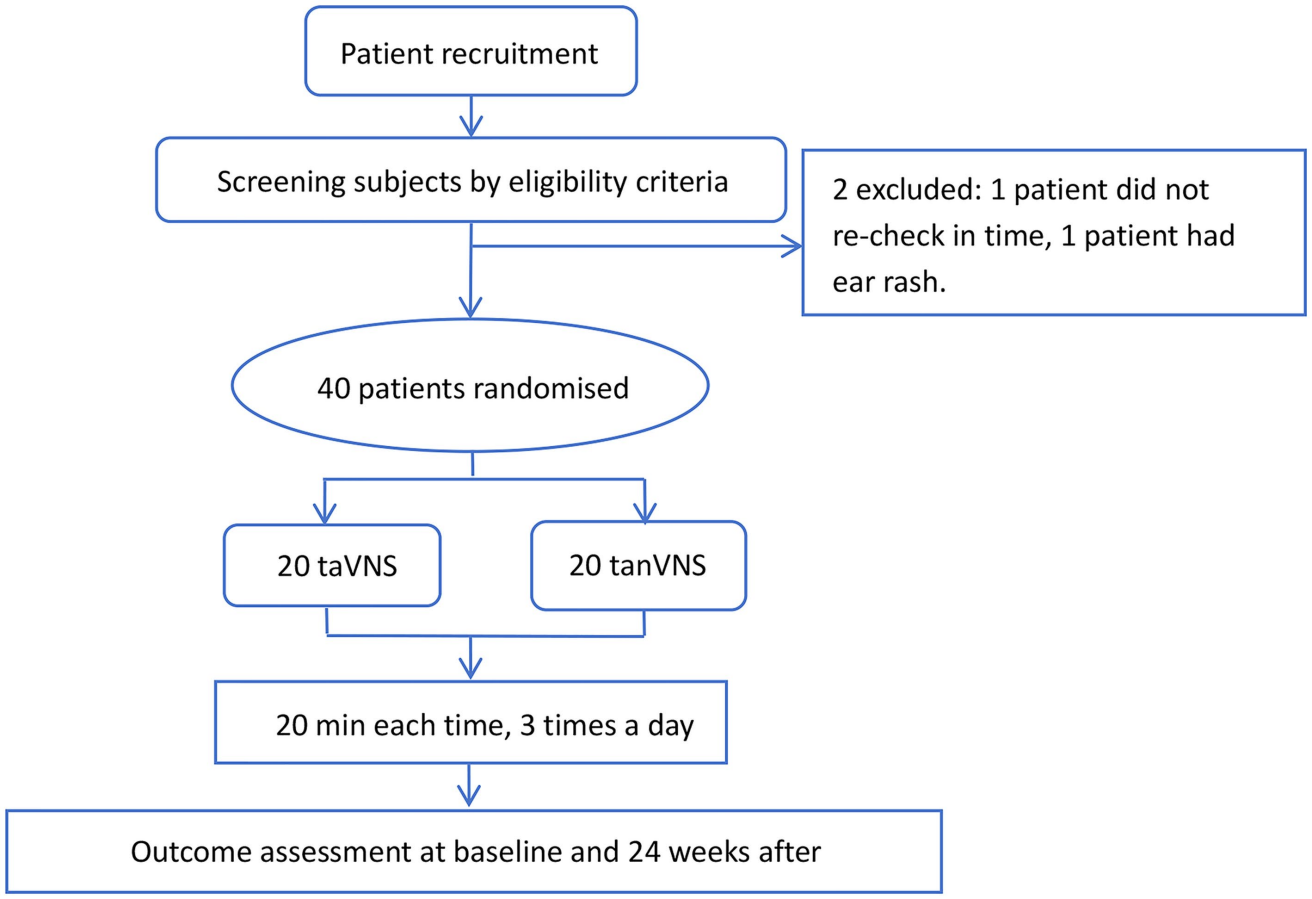
Flowchart of the study.

At the beginning of enrolment, the patients were given a stimulator. The patients were instructed to learn the site of true stimulation or pseudostimulation, depending on the group assignment (the physician did not disclose to the patients whether he or she was assigned to the taVNS group or tanVNS group). Patients were required to keep a detailed diary to report the frequency of seizures and migraine attacks. This trial included a 24-week treatment period. Outcome assessment was performed at baseline and 24 weeks after initiation. A total of 40 patients were ultimately included in the analysis (Figure 1).

### 2.2 Inclusion and exclusion criteria

The inclusion criteria for the patient cohort were as follows: (1) met the diagnostic criteria for epilepsy comorbid with migraine (37), and aged 18 to 65 years (to minimize confounding variables related to age and potential serious diseases); (2) had focal preserved consciousness seizures or focal impaired consciousness seizures (not including focal-to-bilateral tonic–clonic seizures); (3) had an average of two or more seizures per month with mild and short-lived symptoms that did not affect daily life or work; (4) took only valproic acid (VPA) or levetiracetam (LEV) as monotherapy (VPA and LEV, broad-spectrum antiepileptic drugs that are more widely used than other antiepileptic drugs in primary care, can not only treat focal epilepsy but also prevent migraine attacks; as some patients were women of childbearing age, LEV was given) for at least 2 years, with no need to adjust the medication; (5) had migraines for at least 6 months, with at least two migraine attacks per month, but no use of preventive headache medication in the past 3 months; (6) used no psychoactive or vasoactive drugs in the past 3 months; (7) had normal cognitive function (Montreal Cognitive Assessment score equal to or greater than 26 points); and (8) fully understood the trial process, were willing to comply with and participate in this study, and provided informed consent.

The exclusion criteria for the patient cohort were as follows: (1) had mental illness, progressive nervous system disease, brain trauma, or serious physical illness (such as cardiopulmonary diseases); (2) were pregnant or lactating, or planned to become pregnant during the trial period; (3) had migraine caused by other diseases; (4) had other types of headaches, such as tension headaches; (5) had migraine attacks that occurred within 48 h before the trial; (6) had any other chronic pain condition; (7) had severe head deformities or intracranial lesions; or (8) had significant mood disorders, as indicated by an SAS score >50 or SDS score >53.

The withdrawal criteria were as follows: (1) severe adverse reactions after taVNS; (2) participation in other studies that affected brain function; or (3) inability to continue the study for any reason.

### 2.3 Randomization and blinding

Eligible patients who provided consent were given a unique identification number, which was only known by the researchers. Computer-based randomization software was used to randomly divided the patients into the taVNS group and the tanVNS group. For blinding, the group allocations were concealed until the final data analysis report was completed.

### 2.4 Interventions

**Figure 2:**
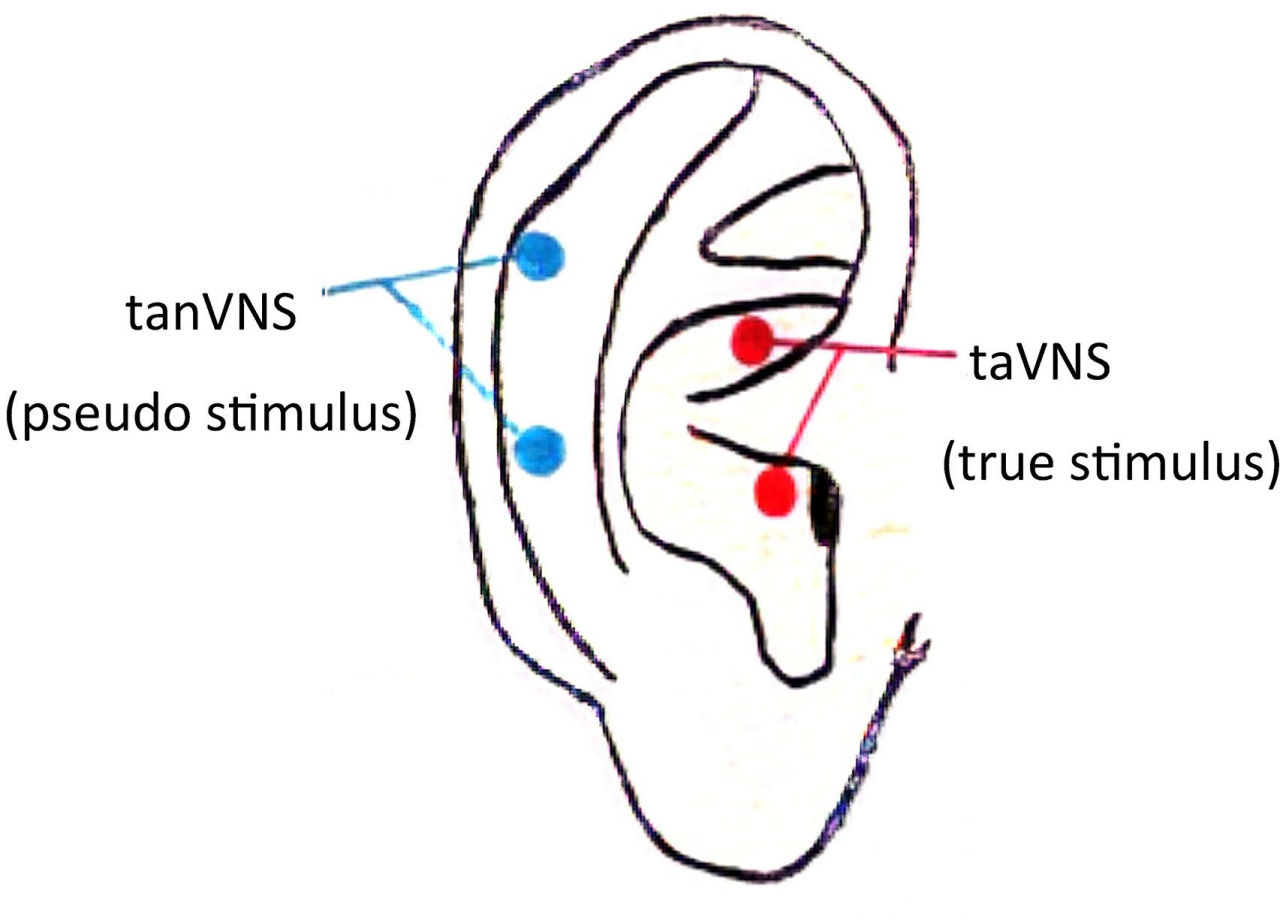
ABVN stimulation site.

A transcutaneous electrical nerve stimulator (TENS sm; Suzhou Medical Appliance Co. Ltd., China) was used in this study. The electrode clamp was designed with three carbon-impregnated silicone electrode tips, one of which acted as the common terminal end and the other two of which were designed to contact the two skin surface points at the triangular fossa of the auricle (taVNS, true stimulus) and the outer ear canal (tanVNS, pseudo stimulus). Only one of the two tips was activated in a single clamp. The stimulation electrodes were made of conductive rubber with a diameter of 5 mm. The specific stimulation site was selected according to a study by Rong et al. (38) (see Figure 2). The stimulation parameters were as follows: frequency of 20 Hz [previous studies revealed that both 1 Hz and 20 Hz can treat migraine (39, 40), and 20–30 Hz has used to treat epilepsy (36); the patients in our study had epilepsy comorbid with migraine, so we chose 20 Hz as the stimulation frequency]; constant voltage, continuous current output; dense-sparse waves; wave width of 0.2 ms; electrical stimulation intensity under the threshold that the patient could tolerate without producing a sharp pain sensation (a sensation similar to the pulsation of blood vessels); range of 4–12 mA; duration of 20 min; three times a day (at approximately 8 in the morning, 12 noon, and 8 at night, eliminating the interference of circadian rhythm); 24-week treatment period. Professional technicians instructed the patients on how to use the stimulators at initiation; then, the patients took the stimulators home and used them regularly as instructed.

### 2.5 Outcome measures

Seizure frequency and migraine attack frequency were determined at baseline and after 24 weeks according to the patients’ diaries. The format and detailed content of the diaries were described in previous studies (41, 42).

Scalp EEG recordings were made using 18 leads [16 channel EEGs (i.e., Fp1/2, F3/4/7/8, C3/4, P3/4, O1/2, and T3/4/5/6) and 2 reference electrodes], and an Australian COMPUMEDICS instrument was used to record a set of digital video EEGs at a sampling rate of 256 Hz. During the examination, the scalp was fully exposed, and conductive cream was applied to the disc electrode. If the impedance was too high, the scalp was wiped with alcohol. Single or dual cameras were used to monitor the behaviour of the patients while they were awake for 2–4 h.

EEG power spectrum analysis was conducted via the ZN500 EEG topography real-time processing system produced by Chengdu Intelligent Electronics Industry Co., Ltd. Four standard EEG frequency bands were selected: the *α* wave (8–13 Hz, 30–50 μV), *β* wave (14–30 Hz, 5–30 μV), *θ* wave (4–7 Hz, 10–40 μV) and *δ* wave (0.5–3.5 Hz, 10–20 μV) bands. The EEG data were processed as follows: the original EEG data were filtered through a bandpass filter (1–46 Hz) to remove interference, and 10 s of the head and tail of the data were removed to eliminate additional interference. After the processing was completed, a doctor who specialized in EEG analysis reviewed the video and selected four 10-s segments that showed intermittent epileptic discharges with few interfering factors (excluding eyeblinks and movements manually) for each patient. After selecting these segments, the power of the *α* wave, *β* wave, *θ* wave and *δ* wave at 16 electrode locations was calculated via computer analysis and statistical processing (fast Fourier transformation). Eventually, the absolute power of each frequency band at 16 electrode locations before and after taVNS or tanVNS treatment was calculated for each group.

Anxiety was measured via the SAS. The statistical index used was the total score. The final score was the crude score multiplied by 1.25.

Depression was measured via the SDS. The statistical index used was the total score. The final score was the crude score multiplied by 1.25.

Quality of life was measured by the Quality of Life in Epilepsy-31 Inventory (QOLIE-31). This scale includes 31 questions across 7 subscales, including seizure worry, overall quality of life, emotional well-being, cognitive, energy/fatigue, medication effects, social function and overall score. First, the raw precoded numeric values of the items were converted to 0–100 point scores (labelled “subtotal”). Next, the subtotal scores for each scale (marked “total”) were summed. Finally, each “total” score was divided by the number of items that the respondent answered within each scale to obtain the “final score.” The possible range of the final score for each scale was 0 to 100 points. Higher scores reflected better quality of life, whereas lower scores indicated worse quality of life. In this study, we used the Chinese version of the QOLIE-31-P because of its satisfactory reliability and validity (Cronbach’s alpha coefficient ranging from 0.627 to 0.898) (43).

### 2.6 Quality control and trial monitoring

Before the initiation of the trial, the researchers formulated an investigator’s brochure, standard operating procedures and detailed research plan. All the staff participated in special training about patient enrolment, completion of the case report form and use of the stimulator. Each time a patient used the stimulator, the researchers engaged in a video call with the patient to ensure the accuracy of electrode placement. Moreover, the stimulation parameters and duration of use were recorded under the supervision of the patient’s family members. The final report was produced in accordance with the Consolidated Standards of Reporting Trials (CONSORT) 2010 guidelines for nonpharmaceutical interventions. The patients were informed that they could receive doctor consultations throughout the entire study period. They were also told they could also receive a physical examination and an auricular vagus nerve stimulator for free if they completed the study. Appendix S1 provides the CONSORT checklist in detail.

### 2.7 Statistical analysis

The raw data from the seizure diary, migraine attack diary, and medical records were entered into Microsoft Excel (Office 2021). All the statistical calculations were performed via SPSS v.27.0. The seizure frequency, migraine attack frequency, EEG power spectrum, SAS score, SDS score and QOLIE-31 score at baseline and after 24 weeks of treatment were compared between the groups. The Kolmogorov–Smirnov test was used to evaluate whether variables followed a normal distribution. Continuous variables are expressed as the means ± SDs or medians ± IQRs, and categorical variables are presented as frequencies and percentages. The normally distributed variables were compared via *t* tests, whereas the nonnormally distributed variables were analysed via non-parametric tests. *p* < 0.05 was considered to indicate statistical significance.

## 3 Results

### 3.1 Clinical characteristics

A total of 40 patients (20 taVNS, 20 tanVNS) were ultimately included in Neurology Outpatient Department. All the patients had focal seizures. The age range of the patients was 21 to 53 years, and the duration of disease was 5–28 years in the taVNS group and 6–34 years in the tanVNS group (Table 1). There were no differences in gender (*p* = 0.75), age (*p* = 0.58) or course of disease (*p* = 0.93) between the two groups. The specific EEG was provided in Supplementary Figure S1.

**Table 1:** Clinical characteristics of patients.

Patients				
		Total	taVNS	tanVNS
Total (numbers)		40	20	20
Sex	Male (numbers)	19	9	10
	Female (numbers)	21	11	10
	*p*	/	0.75	
Age (years)	Range	21–53	21–46	24–53
	Mean ± SD	36.95 ± 6.81	36.35 ± 7.23	37.55 ± 6.50
	*p*	/	0.58	
Course (years)	Range	5–34	5–28	6–34
	Mean ± SD	16.55 ± 6.44	16.55 ± 6.44	16.35 ± 7.02
	*p*	/	0.93	

### 3.2 Evaluation of the therapeutic effect of taVNS

#### 3.2.1 The frequency of migraine attacks and seizures in the taVNS group and tanVNS group of patients before and after treatment

In Table 2, the frequencies of migraine attacks and seizures significantly decreased in the taVNS group from baseline to 24 weeks (migraine attack frequency, *p* = 0.002; seizure frequency, *p* = 0.004).

**Table 2:** The frequency of migraine attacks and seizures in taVNS group and tanVNS group before and after treatment.

Attacks	Groups	Frequency per month (Medians ± IQR)			*p*
		0 W	24 W	Δ = 0 W–24 W	
Migraine	taVNS	3.00 ± 1.75	2.00 ± 1.00	1.50 ± 2.75	0.002
	tanVNS	2.00 ± 0.75	2.00 ± 1.00	0.00 ± 2.00	
Seizure	taVNS	2.00 ± 1.00	1.00 ± 1.00	2.00 ± 0.00	0.004
	tanVNS	2.00 ± 0.75	1.00 ± 0.75	1.00 ± 1.00	

All data were not normally distributed, so non-parametric test (Mann–Whitney *U* test) was used. *p* < 0.05 was considered significant difference. taVNS, transcutaneous auricular vagus nerve stimulation; tanVNS, transcutaneous auricular non-vagus nerve stimulation; W, weeks.

#### 3.2.2 SAS and SDS scores of the taVNS group and tanVNS group before and after treatment

In Table 3, the SAS and SDS scores in the taVNS group significantly decreased from baseline to 24 weeks (*p* < 0.001).

**Table 3:** The scores of SAS and SDS in taVNS group and tanVNS group before and after treatment.

Scores	Groups	Means ± SD or medians ± IQR^a^			*p*
		0 W	24 W	Δ = 0 W–24 W	
SAS	taVNS	47.00 ± 5.00^a^	40.00 ± 5.50^a^	7.00 ± 2.75^a^	<0.001
	tanVNS	46.00 ± 4.75^a^	44.55 ± 2.72	0.30 ± 4.26	
SDS	taVNS	47.85 ± 2.85	38.35 ± 2.87	9.50 ± 2.78	<0.001
	tanVNS	46.10 ± 2.90	43.25 ± 3.27	2.85 ± 4.15	

SAS, Self-Rating Anxiety Scale; SDS, Self-Rating Depression Scale; taVNS, transcutaneous auricular vagus nerve stimulation; tanVNS, transcutaneous auricular non-vagus nerve stimulation; W, weeks.

a Indicates medians ± IQR. Δ SAS scores were not normally distributed so non-parametric test (Mann–Whitney *U* test) was used. Δ SDS scores were normally distributed with homogeneous variance, so independent-samples *t*-test was used. *p* < 0.05 was considered significant difference.

#### 3.2.3 QOLIE-31 scores of the taVNS group and the tanVNS group before and after treatment

In Table 4, there were significant increases in QOLIE-31 overall scores of taVNS group (*p* = 0.028). In the specific sub-items, seizure worry (*p* = 0.020), energy/fatigue (*p* = 0.001), and medication effects (*p* = 0.017) showed significant increases in taVNS group. While overall quality of life (*p* = 0.888), emotional well-being (*p* = 0.761), or social function (*p* = 0.075) did not.

**Table 4:** The score of QOLIE-31 in taVNS group and tanVNS group before and after treatment.

Items	Groups	Medians ± IQR or means ± SD^a^			*p*
		0 W	24 W	Δ = 24 W–0 W	
Seizure worry	taVNS	50.50 ± 15.00	60.15 ± 8.89^a^	2.00 ± 17.17	0.020
	tanVNS	59.28 ± 5.55^a^	60.33 ± 7.10^a^	1.05 ± 7.46^a^	
Overall quality of life	taVNS	45.84 ± 8.33	54.17 ± 18.33	0.00 ± 10.00	0.888
	tanVNS	53.33 ± 10.00	55.00 ± 0.00	3.67 ± 8.84^a^	
Emotional well-being	taVNS	50.00 ± 3.33	51.17 ± 5.11^a^	0.00 ± 5.84	0.761
	tanVNS	50.00 ± 6.66	53.33 ± 3.33	0.00 ± 3.33	
Energy/fatigue	taVNS	47.92 ± 8.33	51.87 ± 6.26^a^	4.17 ± 8.34	0.001
	tanVNS	50.00 ± 3.13	47.92 ± 4.17	0.00 ± 11.45	
Cognitive	taVNS	49.90 ± 2.91^a^	52.47 ± 5.10^a^	0.00 ± 5.55	0.031
	tanVNS	53.40 ± 3.74^a^	52.33 ± 5.02^a^	0.00 ± 7.02	
Medication effects	taVNS	50.84 ± 8.33	58.33 ± 8.38^a^	3.34 ± 13.34	0.017
	tanVNS	56.67 ± 8.33	56.58 ± 7.12^a^	0.50 ± 8.20^a^	
Social function	taVNS	55.93 ± 5.39^a^	60.00 ± 10.50	0.00 ± 4.00	0.075
	tanVNS	57.43 ± 4.86^a^	58.00 ± 2.50	0.23 ± 4.66^a^	
Overall score	taVNS	50.97 ± 1.80	54.40 ± 4.53^a^	2.46 ± 7.30	0.028
	tanVNS	53.80 ± 2.99^a^	54.26 ± 2.82	0.25 ± 3.18^a^	

SAS, Self-Rating Anxiety Scale; SDS, Self-Rating Depression Scale; taVNS, transcutaneous auricular vagus nerve stimulation; tanVNS, transcutaneous auricular non-vagus nerve stimulation; W, weeks; QOLIE-31, quality of life in epilepsy-31 inventory.

a Indicates means ± SD. Non-parametric test (Mann–Whitney U test) was used. *p* < 0.05 was considered significant difference.

### 3.3 Changes in the EEG power spectrum in patients

Table 5 showed the EEG power spectrum (μV^2^) of two groups at 0 W and 24 W, and taVNS reduced the EEG power spectrum in the four frequency bands (*p* < 0.05). Supplementary Tables S1, S2 provide a detailed comparison within groups for the 16 electrode sites.

**Table 5:** Comparison of EEG spectral power (μV^2^) between taVNS group and tanVNS group.

Band	Groups	Sum of 16 electronic sites			*p*
		Medians ± IQR or means ± SD^a^			
		0 W	24 W	Δ = 0 W–24 W	
*δ*	taVNS	274.25 ± 2.20	240.99 ± 1.32^a^	33.20 ± 2.60	<0.001
	tanVNS	272.30 ± 3.68	258.39 ± 0.80^a^	14.65 ± 3.23	
*θ*	taVNS	310.20 ± 1.93	252.05 ± 123.92	57.95 ± 122.40	0.03
	tanVNS	315.61 ± 0.89^a^	284.10 ± 2.22	31.20 ± 1.37	
*α*	taVNS	651.27 ± 1.52^a^	478.08 ± 2.43^a^	173.19 ± 2.80^a^	<0.001
	tanVNS	645.81 ± 2.22^a^	561.20 ± 2.98	84.56 ± 2.88^a^	
*β*	taVNS	867.20 ± 2.00	753.50 ± 1.27	113.80 ± 2.43	<0.001
	tanVNS	845.62 ± 1.28^a^	774.09 ± 2.95^a^	71.53 ± 3.58^a^	

EEG, electroencephalogram; taVNS, transcutaneous auricular vagus nerve stimulation; tanVNS, transcutaneous auricular non-vagus nerve stimulation; W, weeks.

Detailed power spectrum in four frequency bands at 16 electrode locations were in Supplementary Table S1, S2.

a Indicates means ± SD. Δ *δ*, Δ *θ* and Δ *β* were not normally distributed so non-parametric test (Mann–Whitney U test) was used. Δ *α* were normally distributed with homogeneous variance, so independent-samples *t*-test was used. *p* < 0.05 was considered significant difference.

Longitudinal comparison before and after treatment (0 W vs. 24 W): In the patient taVNS group, after 24 weeks of taVNS treatment, there were significant differences in the full frequency band power at each electrode site (except for the O_2_ *δ* segment in the occipital region) (*p* < 0.001, see Supplementary Table S1), and the power was decreased, suggesting that taVNS could help improve the brain power at the full frequency band of 16 electrode sites.

After 24 weeks of sham stimulation in the patient tanVNS group, except for the *θ* band at the FP2 position, the other frequency bands showed significant differences (*p* < 0.05, see Supplementary Table S2), which were also decreased, suggesting that tanVNS may show a certain placebo effect.

These results suggested that the EEG power spectrum did not change only after taVNS, but can also change under tanVNS. Further research is needed in the future to explore whether EEG power spectrum can be used as an indicator for predicting the efficacy of taVNS.

### 3.4 Adverse events

Only one patient showed ear rash at the beginning of the trial, so he withdrew from our study. None of the 40 patients who ultimately participated showed any side effects, and no adverse events were reported during the 24-week follow-up, indicating that the equipment was well tolerated.

## 4 Discussion

By examining data from patients’ epilepsy diaries and migraine diaries, we found that taVNS could reduce the frequency of seizures and migraine attacks in patients with comorbid epilepsy and migraine (see Table 2). Previous studies have confirmed that taVNS can reduce the frequency of seizures in patients with epilepsy or reduce the frequency of migraine attacks in patients with migraine (34, 36, 38, 44–46). Moreover, our study revealed that, according to SAS, SDS and QOLIE-31 scores, taVNS could improve the mood and quality of life of patients with comorbid epilepsy and migraine (see Tables 3, 4). Previous studies have confirmed that taVNS can improve mood and quality of life in patients with epilepsy or migraine (33, 38, 45, 46).

Regarding EEG outcomes, our study revealed that taVNS could reduce the EEG power spectrum of the four frequency bands (*α*, *β*, *δ*, and *θ*) throughout almost the entire brain during the interictal period in patients with comorbid epilepsy and migraine (see Table 5). Previous studies have shown that the power of *θ* and *δ* waves in epilepsy patients is increased (47–50). Additionally, in patients with benign childhood epilepsy with centrotemporal spikes, the absolute power of the *θ*, *δ* and *α* waves is increased (51, 52). Migraine patients have elevated absolute power in almost all frequency bands (53, 54), most significantly in high-frequency *β* waves (55, 56). In addition, migraine patients have increased *θ* relative power (57). These studies revealed an increase in the power of all bands of the EEG spectrum in patients with epilepsy or migraine, and our study revealed that taVNS could decrease the absolute power of these bands and alleviate clinical symptoms. Previous studies have shown that taVNS can decrease *α* activity (58) and *β* activity (59), which is consistent with our findings. On this basis, we speculate that taVNS leads to reduced seizures and migraine attacks by affecting neural pathways and brain region connectivity associated with frequency bands of the EEG power spectrum.

The mechanisms underlying the effects of taVNS on the EEG power spectrum, especially on different frequency bands, have not yet been fully studied. Activation of the NTS (60–62) and LC (63–68) via vagus nerve stimulation is one of the main mechanisms involved. Previous studies revealed that there are direct projections from the ABVN to the NTS in rats, and these projections are considered the anatomical basis of the auriculo-vagus relationship (21, 22). Other brain regions that are modulated include the limbic system, the reticular structure, and the autonomic nervous system in both cerebral hemispheres (64, 69). Through these brain regions, the power of different frequency bands can be modulated. taVNS can reduce the ability of *α* activity to suppress the interference effects of irrelevant information in prefrontal cortices (70–72), which presumably reduces abnormal discharges in patients with frontal lobe epilepsy. Increased *β* activity is significantly associated with epilepsy and migraine (73), and it is speculated that taVNS can reduce *β* activity by modulating cortico-thalamo-cortical (CTC) networks (74). taVNS can significantly modulate the activity/connectivity of brain regions associated with the central vagus nerve pathway and pain modulation system, including the DMN and brainstem areas (LC, raphe nuclei, parabrachial nucleus, and solitary nucleus) (75). Functional connectivity analysis revealed that taVNS can increase the connectivity between the motor-related thalamic subregion and anterior cingulate cortex/medial prefrontal cortex and decrease the connectivity between the occipital cortex-related thalamic subregion and the postcentral gyrus/precuneus (34). *δ* waves are essentially absent in the awake state under physiological conditions, and they are highly pronounced when subcortical brain damage occurs (76, 77). Our study revealed a decrease in *δ* activity in patients after taVNS, so we speculated that taVNS may regulate the thalamic-cortex circuit to attenuate harmful brain activity, such as seizures and migraine attacks, by decreasing *δ* activity. However, some studies have shown that taVNS increases *δ* activity and *θ* activity (78–80), which is inconsistent with our findings. Therefore, more studies are needed to explore the effects of taVNS on different frequency bands of the EEG power spectrum and the related mechanisms.

Pseudostimulation (tanVNS) could also decrease the EEG power spectrum (see Supplementary Table S2), but the decrease was less than that caused by taVNS (see Table 5). Although the stimulation of a very small number of ABVN peripheral branches by the pseudostimuli (38) may have slightly decreased the EEG power spectrum, the degree and range of stimulation were not enough to reduce the incidence of clinical seizures and migraine attacks in patients (see Table 2), which suggests that the selection of the ABVN stimulation site is very important for the clinical treatment effect (see Figure 2); specifically, the triangular fossa of the auricle should be chosen, as it has a more dense ABVN distribution (38).

### 4.1 Limitations

The sample size of this study was small so studies with larger sample sizes should be considered in the future. In our study, all patients with comorbid epilepsy and migraine received only monotherapy with the antiseizure medication VPA (25 patients used; 0.5 g bid) or LEV (15 patients used; 0.5 g bid). Owing to the small number of patients included, medication status could not be analysed as a covariate. We used only EEG power spectrum as an objective indicator of functional neuroimaging; therefore, in future studies, fMRI and EEG mathematical model analyses (such as the seizure detection model) could be used to further elucidate the mechanism underlying the effect of taVNS on brain networks. Our study included patients with epilepsy comorbid with migraine but did not include patients with only one of the disorders (epilepsy or migraine alone). The mechanism of epilepsy comorbid with migraine is complex (81), so comparisons of patients with only one of these diseases and both diseases are needed to identify the relationship between epilepsy and migraine. In addition, the relationship between clinical symptom relief and changes in the EEG power spectrum must be explored further.

## 5 Conclusion

The efficacy of taVNS in patients with epilepsy or migraine has been studied previously, but studies in patients with epilepsy comorbid with migraine are lacking. This study is the first of use taVNS in patients with comorbid epilepsy and migraine. As a noninvasive adjunctive therapy, taVNS can reduce the EEG power spectrum in patients with comorbid epilepsy and migraine. Patients’ epilepsy diaries and migraine diaries suggested that taVNS could reduce the frequency of seizures and migraine attacks. According to SAS, SDS, and QOLIE-31 scores, taVNS could improve the mood and quality of life of patients with comorbid epilepsy and migraine. In the future, further exploration of the mechanism underlying the effect of taVNS, such as changes in brain networks, would be useful to provide more treatment options for patients.

## Data Availability

The original contributions presented in the study are included in the article/Supplementary material, further inquiries can be directed to the corresponding author.
