# Supplementary material for: The effects of transcutaneous auricular vagus nerve stimulation in epilepsy comorbid with migraine on the EEG power spectrum: a randomized controlled trial
Source: Front Neurol. 2025 Dec 12;16:1694455. doi: 10.3389/fneur.2025.1694455 (PMC12740757; doi:10.3389/fneur.2025.1694455)
Supplement: Supplementary file 2 [file Table_2.docx]

Longitudinal comparison before and after treatment (0W vs. 24W) : In the patient taVNS group, after 24 weeks of taVNS treatment, there were significant differences in the full frequency band power at each electrode site (except for the O2δ segment in the occipital region) (*P* < 0.001, see Table S1), and the power was decreased, suggesting that taVNS could help improve the brain power at the full frequency band of 16 electrode sites.

After 24 weeks of sham stimulation in the patient tanVNS group, except for the θ band at the FP2 position, the other frequency bands showed significant differences (*P* < 0.05, see Table S2), which were also decreased, suggesting that tanVNS may show a certain placebo effect.

These results suggested that the EEG power spectrum did not change only after taVNS, but can also change under tanVNS. Further research is needed in the future to explore whether EEG power spectrum can be used as an indicator for predicting the efficacy of taVNS.

**Table S1. Comparison of EEG spectral power (μV^2^) at 0W and 24W in taVNS group**

|  | **Electrode** | **Band** | **0W(N=20)**  **Medians ± IQR** | **24W(N=20)**  **Medians ± IQR** | ***P*** |
| --- | --- | --- | --- | --- | --- |
| **Frontal pole** | FP1 | δ | 18.2±0.10 | 15.35±0.25 | <0.001 |
|  |  | θ | 21.2±0.20 | 14.45±0.60 | <0.001 |
|  |  | α | 32±0.20 | 24.6±0.20 | <0.001 |
|  |  | β | (means ± SD)  54.55±0.23 | (means ± SD)  49.31±0.15 | <0.001 |
|  | FP2 | δ | 18.55±0.10 | 15.2±0.10 | <0.001 |
|  |  | θ | 20.7±0.40 | 13.9±123.18 | <0.001 |
|  |  | α | 31.9±1.10 | 25.5±0.60 | <0.001 |
|  |  | β | 53.1±0.40 | 20.1±0.10 | <0.001 |
| **Frontal region** | F3 | δ | 17.3±0.18 | 16.2±0.30 | <0.001 |
|  |  | θ | 19.4±0.18 | 14.6±0.20 | <0.001 |
|  |  | α | 34.2±0.00 | 27.5±1.00 | <0.001 |
|  |  | β | 53.7±0.80 | 49.15±0.10 | <0.001 |
|  | F4 | δ | 17.6±0.10 | 16.1±0.10 | <0.001 |
|  |  | θ | 18.7±0.20 | 15.2±0.18 | <0.001 |
|  |  | α | 35.1±0.00 | 28.2±0.00 | <0.001 |
|  |  | β | 53.9±0.40 | 48.5±0.20 | <0.001 |
| **Temporal region** | F7 | δ | 16.2±0.10 | 15.3±0.30 | <0.001 |
|  |  | θ | 19.4±0.10 | 13.9±0.07 | <0.001 |
|  |  | α | 29.4±0.50 | 21.6±0.10 | <0.001 |
|  |  | β | 54.5±0.20 | 47.1±0.20 | <0.001 |
|  | F8 | δ | 16±0.53 | 14.4±0.38 | <0.001 |
|  |  | θ | 18.5±0.1 | 14.2±0.6 | <0.001 |
|  |  | α | 30.2±0.40 | 22.3±0.38 | <0.001 |
|  |  | β | 55.2±0.10 | 47.6±0.30 | <0.001 |
|  | T3 | δ | 15.9±0.10 | 13.9±0.28 | <0.001 |
|  |  | θ | 18.65±0.10 | 12.9±0.10 | <0.001 |
|  |  | α | 25.2±3.40 | 19.4±0.38 | <0.001 |
|  |  | β | 53.4±0.30 | 47.95±0.20 | <0.001 |
|  | T4 | δ | 15.45±0.10 | 12.9±0.30 | <0.001 |
|  |  | θ | 19.2±0.10 | 13.2±0.10 | <0.001 |
|  |  | α | 25.6±0.70 | 19.3±0.00 | <0.001 |
|  |  | β | 53.6±0.25 | 47.6±0.70 | <0.001 |
|  | T5 | δ | 16.85±0.27 | 15.3±0.10 | <0.001 |
|  |  | θ | 15.6±0.20 | 14±0.17 | <0.001 |
|  |  | α | 43.2±1.05 | 31.7±0.80 | <0.001 |
|  |  | β | 54.3±0.30 | 48.8±0.20 | <0.001 |
|  | T6 | δ | 17.45±0.48 | 16.2±0.20 | <0.001 |
|  |  | θ | 16.2±0.18 | 14.5±0.40 | <0.001 |
|  |  | α | 46.8±0.40 | 33.7±0.50 | <0.001 |
|  |  | β | 54.2±0.10 | 49.15±0.40 | <0.001 |
| **Central core** | C3 | δ | 16.9±0.18 | 15.1±0.15 | <0.001 |
|  |  | θ | 20.3±0.10 | 13.9±0.10 | <0.001 |
|  |  | α | 45.2±2.40 | 34.2±0.53 | <0.001 |
|  |  | β | 54.7±0.20 | 50±0.18 | <0.001 |
|  | C4 | δ | 17.2±0.17 | 13.8±0.80 | <0.001 |
|  |  | θ | 20.8±0.20 | 16±0.67 | <0.001 |
|  |  | α | 46.7±0.00 | 35.1±0.20 | <0.001 |
|  |  | β | 54.75±0.40 | 49.1±0.20 | <0.001 |
| **Parietal region** | P3 | δ | 17.9±0.10 | 15±0.10 | <0.001 |
|  |  | θ | 21.1±0.48 | 14.5±0.15 | <0.001 |
|  |  | α | 56.2±2.47 | 39.8±0.07 | <0.001 |
|  |  | β | 55.9±0.00 | 50±0.28 | <0.001 |
|  | P4 | δ | 18.4±1.05 | 14.85±0.70 | <0.001 |
|  |  | θ | 21.2±0.07 | 14.9±0.10 | <0.001 |
|  |  | α | 56.9±0.10 | 36.9±0.70 | <0.001 |
|  |  | β | 54.8±0.80 | 47.9±0.17 | <0.001 |
| **Occipital region** | O1 | δ | 16.3±0.30 | 14.6±0.20 | <0.001 |
|  |  | θ | 19.2±0.07 | 13.1±0.45 | <0.001 |
|  |  | α | 54.9±0.52 | 39±0.40 | <0.001 |
|  |  | β | 53.9±0.10 | 50.2±0.20 | <0.001 |
|  | O2 | δ | 17.75±0.40 | 17.55±0.08 | 0.46 |
|  |  | θ | 19.4±1.10 | 38.95±1.78 | <0.001 |
|  |  | α | 56.60±0.37 | 40.93±0.73 | <0.001 |
|  |  | β | 53.75±0.60 | 51.15±0.30 | <0.001 |

The two groups (0W and 24W) of data in the β band of FP1 electrode were in accordance with the normal distribution and homogeneity of variance, and the paired *t*-test was performed (df=19). The rest of the data did not meet the normal distribution, and two related samples nonparametric test was performed. *P* < 0.05 was considered significant difference. N, numbers; taVNS, transcutaneous auricular vagus nerve stimulation; W, weeks

**Table S2. Comparison of EEG spectral power(μV^2^) at 0W and 24W in tanVNS group**

|  | **Electrode** | **Band** | **0W(N=20)**  **Medians ± IQR** | **24W(N=20)**  **Medians ± IQR** | ***P*** |
| --- | --- | --- | --- | --- | --- |
| **Frontal pole** | FP1 | δ | 18.2±0.20 | 17.6±0.50 | <0.001 |
|  |  | θ | 20.3±0.77 | 18.5±0.10 | <0.001 |
|  |  | α | 31.5±0.35 | 26.4±0.20 | <0.001 |
|  |  | β | 18.2±0.20 | 17.6±0.50 | <0.001 |
|  | FP2 | δ | 19.2±0.00 | 18.2±0.20 | <0.001 |
|  |  | θ | 20.7±0.20 | 14.9±0.00 | 1.00 |
|  |  | α | 30.2±0.00 | 26.9±0.40 | <0.001 |
|  |  | β | 19.2±0.00 | 18.2±0.20 | <0.001 |
| **Frontal region** | F3 | δ | 19±0.20 | 18.1±0.10 | <0.001 |
|  |  | θ | 19.2±0.25 | 16.7±0.17 | <0.001 |
|  |  | α | 33.1±0.70 | 28.9±0.30 | <0.001 |
|  |  | β | 19±0.20 | 18.1±0.10 | <0.001 |
|  | F4 | δ | 17.2±0.90 | 17.1±0.00 | 0.007 |
|  |  | θ | 19.5±0.10 | 18.15±0.20 | <0.001 |
|  |  | α | 38.15±0.25 | 35.4±0.30 | <0.001 |
|  |  | β | 17.2±0.90 | 17.1±0.00 | 0.007 |
| **Temporal region** | F7 | δ | 16.2±0.00 | 16.05±0.10 | <0.001 |
|  |  | θ | 21.2±0.20 | 18.5±0.20 | <0.001 |
|  |  | α | 29.4±0.38 | 27.55±0.70 | <0.001 |
|  |  | β | 16.2±0.00 | 16.05±0.10 | <0.001 |
|  | F8 | δ | 15.3±0.52 | 14±0.28 | <0.001 |
|  |  | θ | 19.2±0.20 | 17.9±0.10 | <0.001 |
|  |  | α | 30.2±0.07 | 28.5±0.10 | <0.001 |
|  |  | β | 15.3±0.52 | 14±0.28 | <0.001 |
|  | T3 | δ | 14.25±0.30 | 13.1±0.40 | <0.001 |
|  |  | θ | 19.7±0.28 | 14.4±0.50 | <0.001 |
|  |  | α | 27.5±0.30 | 25±0.70 | <0.001 |
|  |  | β | 14.25±0.30 | 13.1±0.40 | <0.001 |
|  | T4 | δ | 14.5±0.10 | 14.15±0.17 | <0.001 |
|  |  | θ | 18.4±0.30 | 16.9±0.20 | <0.001 |
|  |  | α | 29.3±0.30 | 26.3±0.30 | <0.001 |
|  |  | β | 14.5±0.10 | 14.15±0.17 | <0.001 |
|  | T5 | δ | 16.1±0.10 | 15.75±1.10 | <0.001 |
|  |  | θ | 15.7±0.08 | 16.2±0.20 | <0.001 |
|  |  | α | 46.15±0.10 | 41.5±0.40 | <0.001 |
|  |  | β | 16.1±0.10 | 15.75±1.10 | <0.001 |
|  | T6 | δ | 17.3±0.25 | 17.1±0.38 | <0.001 |
|  |  | θ | 17.2±0.25 | 13.4±0.18 | <0.001 |
|  |  | α | 45.9±0.30 | 40.6±0.40 | <0.001 |
|  |  | β | 17.3±0.25 | 17.1±0.38 | <0.001 |
| **Central core** | C3 | δ | 16.25±0.10 | 14±0.17 | <0.001 |
|  |  | θ | 21.2±0.20 | 18.1±0.10 | <0.001 |
|  |  | α | 39.9±0.10 | 32.7±0.17 | <0.001 |
|  |  | β | 16.25±0.10 | 14±0.17 | <0.001 |
|  | C4 | δ | 17.5±0.00 | 16.3±0.10 | <0.001 |
|  |  | θ | 20.4±0.10 | 20±0.20 | <0.001 |
|  |  | α | 44.6±0.10 | 39.4±0.25 | <0.001 |
|  |  | β | 17.5±0.00 | 16.3±0.10 | <0.001 |
| **Parietal region** | P3 | δ | 18.2±0.80 | 17.2±0.45 | <0.001 |
|  |  | θ | 19.7±0.63 | 16.4±0.00 | <0.001 |
|  |  | α | 59.6±2.40 | 49.6±0.60 | <0.001 |
|  |  | β | 18.2±0.80 | 17.2±0.45 | <0.001 |
|  | P4 | δ | 19±0.10 | 17.6±0.30 | <0.001 |
|  |  | θ | 22.65±0.50 | 20.1±0.20 | <0.001 |
|  |  | α | 55.3±0.30 | 48.9±1.00 | <0.001 |
|  |  | β | 19±0.10 | 17.6±0.30 | <0.001 |
| **Occipital region** | O1 | δ | 16.5±0.33 | 15.9±0.20 | <0.001 |
|  |  | θ | 20.85±0.50 | 18.6±0.25 | <0.001 |
|  |  | α | 50.35±0.72 | 40.1±0.60 | <0.001 |
|  |  | β | 16.5±0.33 | 15.9±0.20 | <0.001 |
|  | O2 | δ | 17.55±0.80 | 16.6±0.18 | <0.001 |
|  |  | θ | 19.25±0.38 | 26.7±0.27 | <0.001 |
|  |  | α | 18.2±0.20 | 45.1±1.92 | <0.001 |
|  |  | β | 20.3±0.77 | 16.6±0.18 | <0.001 |

All data did not meet the normal distribution, and two related samples nonparametric test was performed. *P* < 0.05 was considered significant difference. N, numbers; tanVNS, transcutaneous auricular non-vagus nerve stimulation; W, weeks
