# Supplementary figures and images for: The effects of transcutaneous auricular vagus nerve stimulation in epilepsy comorbid with migraine on the EEG power spectrum: a randomized controlled trial
Source: Front Neurol. 2025 Dec 12;16:1694455. doi: 10.3389/fneur.2025.1694455 (PMC12740757; doi:10.3389/fneur.2025.1694455)

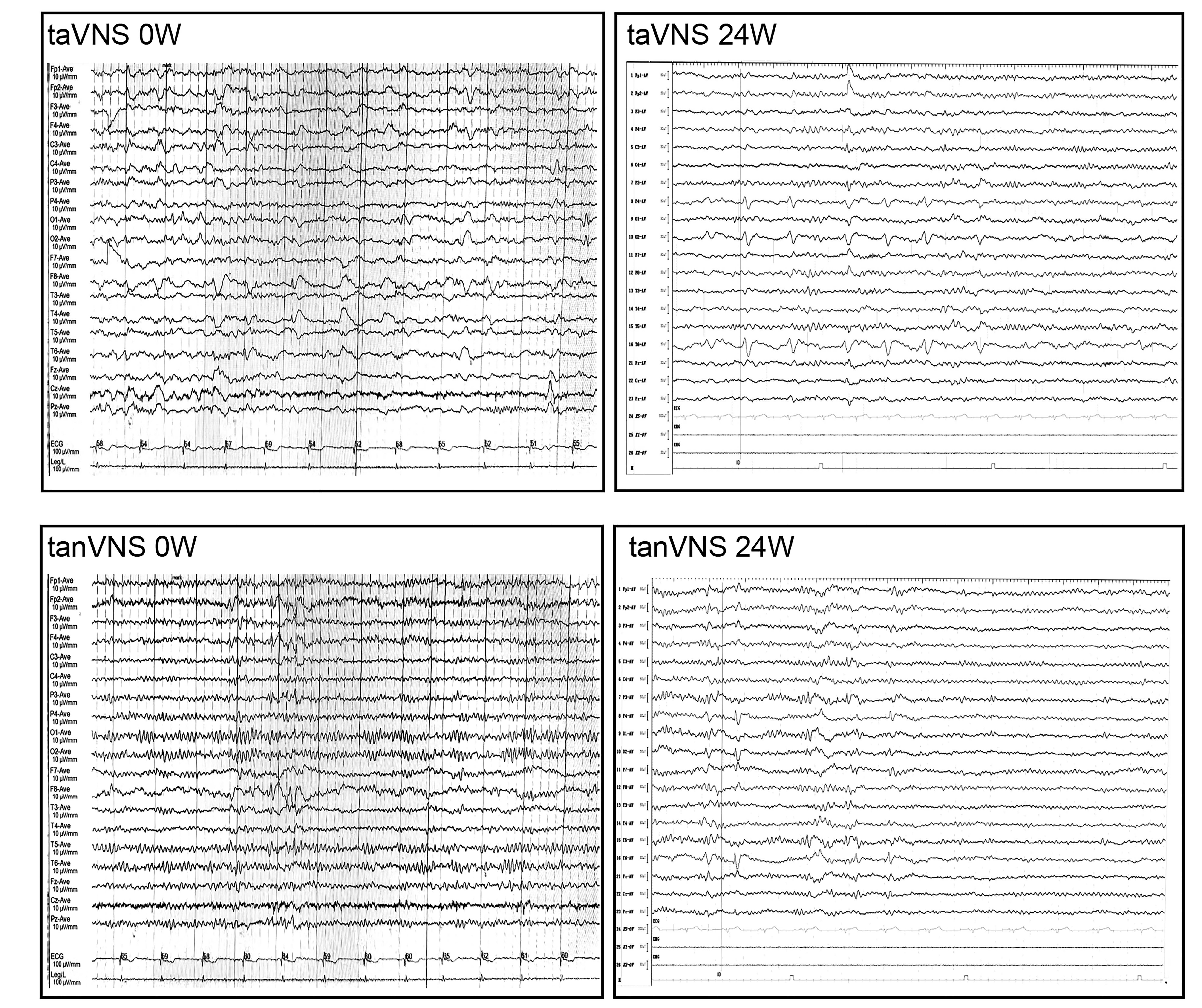

Supplement: Supplementary file 3 [file Image_1.tif]

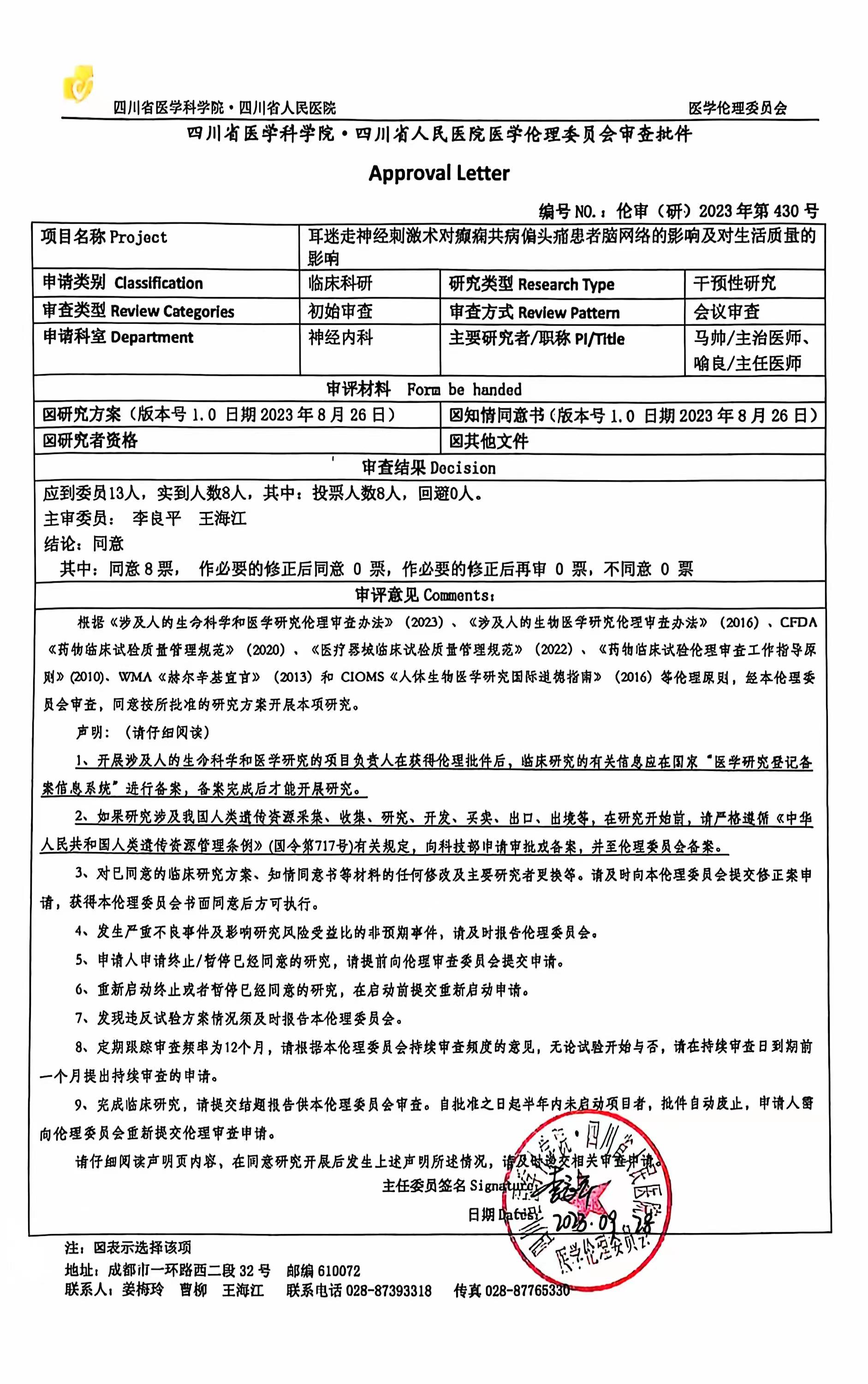

Supplement: Supplementary file 4 [file Image_2.jpg]
